# Self-reported health related quality of life in children and adolescents with an eating disorder

**DOI:** 10.1186/s40359-024-01684-y

**Published:** 2024-04-29

**Authors:** A. Wever, E. van Gerner, J.C.M Jansen, B. Levelink

**Affiliations:** 1 Department of Paediatrics, Maastricht University Medical Centre (MUMC+), P. Debyelaan 25, 6229 HX Maastricht, The Netherlands; 2 Department of Primary care, Radboud University Medical Centre, Geert Grooteplein Zuid 10, 6525 GA Nijmegen, The Netherlands; 3 Department of Child and Youth Psychiatry, Postweg 88, 5915 HB De Mutsaersstichting, Venlo, The Netherlands

**Keywords:** Health Related Quality of Life, Children, Adolescents, Eating disorder, Anorexia nervosa, Avoidant/Restrictive food intake disorder

## Abstract

**Background:**

Eating disorders in children and adolescents can have serious medical and psychological consequences. The objective of this retrospective quantitative study is to gain insight in self-reported Health Related Quality of Life (HRQoL) of children and adolescents with a DSM-5 diagnosis of an eating disorder.

**Method:**

Collect and analyse data of patients aged 8–18 years, receiving treatment for an eating disorder. At the start and end of treatment patients completed the KIDSCREEN-52, a questionnaire measuring HRQoL.

**Results:**

Data of 140 patients were analysed. Children diagnosed with Anorexia Nervosa, Bulimia Nervosa, and Other Specified Feeding or Eating Disorder all had lower HRQoL on multiple dimensions at the start of treatment, there is no statistically significant difference between these groups. In contrast, patients with Avoidant Restrictive Food Intake Disorder only had lower HRQoL for the dimension Physical Well-Being. HRQoL showed a significant improvement in many dimensions between start and end of treatment, but did not normalize compared to normative reference values of Dutch children.

**Conclusion:**

The current study showed that self-reported HRQoL is low in children with eating disorders, both at the beginning but also at the end of treatment. This confirms the importance of continuing to invest in the various HRQoL domains.

## Background

Eating disorders in children and adolescents can have serious medical and psychological consequences and rank 12th on the list of physical and mental conditions amongst woman aged 15–19 years in high-income countries when looking at the global burden of disease [1, 2]. The estimated lifetime prevalence of Anorexia Nervosa (AN) in woman is 1–4% and 1–2% for Bulimia Nervosa (BN), and the epidemiology is changing, with increasing rates of eating disorders in younger children, boys and minority groups [2, 3].

The past two decades research on health-related quality of life (HRQoL) in patients with an eating disorder has increased [4–8]. HRQoL is a subjective evaluation of the overall health of an individual, as well as the health of underlying subdimensions of physical, psychological and social functioning [9]. Most studies have been conducted in adults and a recent review and meta-analysis both show that eating disorders are associated with significant impaired HRQoL compared with the healthy population [10, 11]. To our knowledge only one other study evaluated the impact of eating disorders on HRQoL in children and adolescents. Jenkins et al. looked at the impact of eating disorders in a group of adolescents seeking treatment for AN, BN or eating disorder not otherwise specified (EDNOS) [6]. This study reported a poorer HRQoL measured with the SF-36 Health Survey in adolescents with an eating disorder compared with adolescent norms for the Swedish population [6]. Two studies included both children and adolescents. Weigel et al. examined the association between disorder specific factors, comorbidity and HRQoL in anorexia nervosa in adolescents and adults. HRQoL was measured using the visual analogue scale (EQ-VAS) a generic scale that does not look to different HRQoL domains [12]. Ackard et al. assessed quality of life in patients diagnosed with an eating disorder, mean age at initial assessment was 20.6 years (SD 5 8.3 years), with a range of 12–53 years. Children were not assessed separatly. Other studies in children and adolescents focused on disordered eating behaviours, but not diagnosed eating disorders [4]. A review of population-based studies showed that disordered eating attitudes and behaviours were associated with lower HRQoL in children and adolescents [9]. Herpertz-Dahlmann and colleagues found a poorer HRQoL in adolescents with self-reported disordered eating, and an association between eating disorder symptoms and psychopathology [13].

Because treating an eating disorder encompasses more than weight gain alone it is important to know the possible impact of an eating disorder on HRQoL [14]. As there are still few studies on self-reported HRQoL in children and adolescents with a diagnosed eating disorder, the primary aim of this study is to gain more insight in the different domains of self-reported HRQoL in a clinical sample of children and adolescents with a DSM-5 diagnosis of an eating disorder at the beginning of treatment. In addition, changes of HRQoL between start and end of treatment were evaluated to determine whether treatment influences HRQoL and if so which domains.

## Method

### Participant

Data of patients who were diagnosed conform the Diagnostic and Statistical Manual of Mental Disorders (DSM) -IV-TR/DSM-5 criteria for an eating disorder, and receiving treatment between November 2006 and April 2019 at The Mutsaersstichting were used [15, 16]. The Mutsaersstichting is a mental healthcare institute specialised in eating disorders in the Netherlands where children between 0 and 18 years receive both in- and outpatient treatment. At first presentation, every patient received an extensive consultation with a child and youth psychologist, a child and youth psychiatrist and a paediatrician. Based on this information DSM-IV-TR and DSM-5 classification were made. Patients diagnosed before 2014 were rediagnosed using the DSM-5 classification, especially using the new criteria for Avoidant Restrictive Food Intake Disorder (ARFID). Subsequently, a personalized treatment plan was presented to the family. Treatment always consisted of a combination of family-based treatment, individual treatment, group treatment and physical follow-up. Data from patients who met the DSM-5 diagnosis for AN, BN, ARFID, Binge Eating Disorder (BED), or OSFED were considered eligible for analyses. Because the study specifically focused on self-reported HRQoL, only data of children between the ages of 8 and 18 were included, since for younger children the parents completed the HRQoL questionnaire. Children and adolescents who only had HRQoL reports completed by the parents were excluded. Ethical approval was obtained from the medical ethics committee of the Maastricht University Medical Centre.

### Procedure

As part of the Routine Outcome Monitoring the KIDSCREEN-52 questionnaire was sent to every patient who sought treatment for an eating disorder at the Mutsaersstichting. Baseline characteristics and clinical data were collected at the start and end of treatment. At first consultation, patient characteristics including age, sex, underlying diseases, eating attitudes and behaviours, compensatory behaviour and sociodemographic data were obtained. Heart rate and blood pressure were measured with an oscillometric blood pressure machine and evaluated according to the Clinical Practice Guidline of the American Acadamy of Pediatrics [17]. In addition, a full physical examination was performed. Body Mass Index (BMI) was calculated from measured weight and height [18]. Growth charts designed by the Dutch organization for applied scientific research (TNO) were used to determine height for age (standard deviation, SD) and weight for height (SD) [19, 20]. At the end-evaluation data was collected concerning most recent height, weight, BMI and eating attitudes and behaviours.

### Measures

The KIDSCREEN-52 is a validated self-report questionnaire for measuring HRQoL in European children between 8 and 18 years old [21–25]. It consists of 52 questions, divided into 10 dimensions: Physical Well-being, Psychological Well-being, Moods and Emotions, Self-Perception, Autonomy, Parent Relations and Home Life, Social Support and Peers, School Environment, Social Acceptance (Bullying), and Financial Resources. The KIDSCREEN-52 uses 5-point Likert scale responses, within each different dimension the results are converted into a Rasch scale. Cronbach–alpha’s vary between 0.77 and 0.89 [25]. The results are transformed to a t-score, giving the children in the total reference population a mean t-score of 50 with a SD of 10. Specific reference populations are made by country, gender and age groups. The results of this study are compared with the validated normative reference values of Dutch children in the age between 8 and 18 years old [25]. Ulrike Ravens-Sieberer defined a mean t-score 0.5 SD below the mean t-score of the specific referential population of a country as a low HRQoL and a t-score 0.5 SD above the mean t-score of the referential population as high HRQoL [25].

### Data analysis

All statistical analyses were performed using IBM SPSS Statistics version 25 [26]. The Mann-Withney U, χ^2^, and fisher exact test were used to determine whether there were statistical differences between all the children and adolescents included in this study and the children who completed the questionnaire at intake and end-evaluation. Paired *t* test was used to test for statistically significant differences in HRQoL between start- and end of treatment. To test the differences in t-score on the KIDSCREEN-52 stratified for DSM-classification, a one-way ANOVA and Welch test was done, with the Tukey’s Test as a post-hoc analysis. Statistically significance was considered when the result had a *p* value of < 0.05. Univariate regression analysis was done in the group diagnosed with AN to test whether there is an association between HRQoL and BMI, BMI SD, age, excessive exercise and binge eating. Since purging only occurred in four patients this could not be included in the analysis. Other DSM-5 diagnoses where not included due to small subgroup sample size.

## Results

### Baseline characteristics

**Fig. 1:**
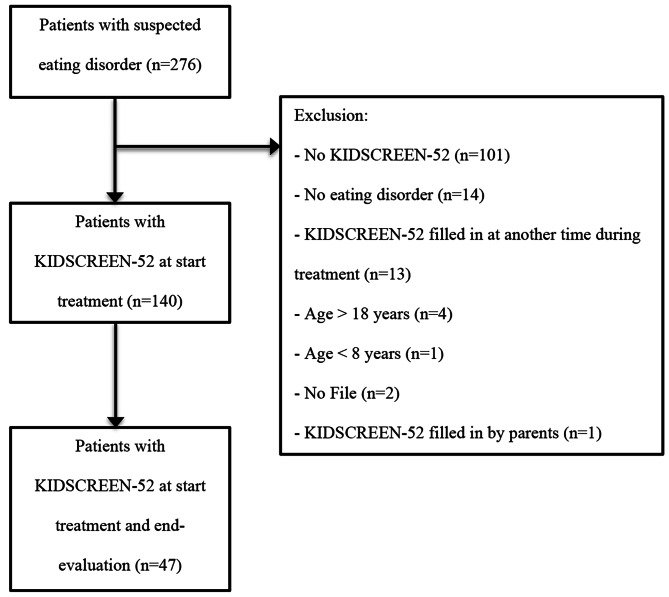
Study Flow

Data of 276 patients were analysed of which 140 were found eligible for this study (Fig. 1). Baseline characteristics are presented in Table 1. The total population consisted primarily of female children and adolescents (*n* = 119; 85%) with a mean age of 15.0 years ranging from 8 to 18 years. Almost half of the population was classified as AN (*n =* 68; 48.6%). The mean weight for children with AN (*n* = 67) was 44.1 kg (minimal weight 25 kg– maximal weight 59 kg) with a mean weight SD of -1.6 and mean BMI of 15.9 kg/m^2^ (minimal BMI 11.9 kg/m^2^– maximal BMI 19.9 kg/m^2^). Children with ARFID had a mean weight of 31.9 kg (minimal weight 18 kg– maximal weight 105 kg), mean weight SD– 0.6, mean BMI 15.9 kg/m^2^ (minimal BMI 12.1 kg/m^2^– maximal BMI 36.3 kg/m^2^). Only two patients were diagnosed with BED, this was too small a sample size to be included in results stratified for the DSM-5 criteria. No significant differences were found in the baseline characteristics between children who completed the KIDSCREEN-52 only at the beginning of treatment, and those who completed the questionnaire both at the start and end-evaluation (*n* = 47), except for psychiatric co-morbidities (*X*^*2*^ (1) = 4.97; *p* = 0.026). Even though the effect size for this finding, Cramer’s V = 0.188, was weak, due to the known association between psychiatric co-morbidities and eating disorder symptoms, a comparison between HRQoL at the beginning and end of treatment was only made within the group of 47 patients that completed both questionnaires [13, 27, 28].

**Table 1:** Baseline Characteristics

	KIDSCREEN-52 at start treatment (*n* = 140)				KIDSCREEN-52 at start- and end treatment (*n* = 47)			
	Start treatment		End-evaluation^a^		Start treatment		End-evaluation^b^	
	Median [IQR]		Median [IQR]		Median [IQR]		Median [IQR]	
Age years	15	[14.7–16.2]	16.6	[14.7–17.8]	14.5	[13.1–15.6]	16.3	[14.6–17.0]
Weight kg	45.6	[38.6–51.0]	51.1	[43.8–57.2]	45	[35.7–51.8]	51.1	[44.5–58.2]
Weight SD	-1.1	[-2.1- -0.3]	-0.7	[-1.4-0.1]	-0.9	[− 2.3- -0.3]	-0.8	[-1.7-0.2]
Length cm	165	[160–169]	165	[161–170]	165	[161–169]	167	[162–171]
Length SD	-0.3	[-1.0-0.3]	-0.62	[-1.3-0.1]	-0.1	[-0.9-0.4]	-0.4	[-1.4–0.0]
BMI kg/m^2^	16.8	[14.7–18.6]	18.8	[16.6–20.5]	16.7	[13.9–18.7]	18.4	[17.3–20.3]
BMI SD	-1.2	[-2.4- -0.2]	-0.52	[-1.5-0.3]	-1.4	[-3.1- -0.2]	-0.6	[-1.5-0.2]
Treatment duration months	15	[8–23]			16	[9–21]		
	*Amount (%)*				*Amount (%)*			
Sex: Female	119 (85)				41 (87.2)			
DSM-classification								
AN	68 (48.6)				23 (48.9)			
BN	13 (9.3)				2 (4.3)			
BED	2 (1.4)				1 (2.1)			
ARFID	28 (20.0)				8 (17.0)			
OSFED	29 (20.7)				13 (27.7)			
Number of previous treatments								
0	64 (45.7)				24 (40.4)			
1–2	65 (46.4)				19 (40.4)			
>2	12 (8.6)				4 (8.5)			
Vomiting	27 (19.3)				7 (14.9)			
Laxantia	7 (0.1)				0 (0)			
Excessive exercise	48 (34.3)				17 (23.4)			
Binge eating	31 (22.1)				11 (23.4)			
Psychiatric co-morbidity^c^	44 (31.4)				10 (21.1)			

Note. BMI = Body Mass Index, AN = anorexia nervosa, BN = bulimia nervosa, BED = binge eating disorder, ARFID = avoidant/restrictive food intake disorder, OSFED = other specified feeding or eating disorder. ^a^data only available of 119 patients. ^b^data only available of 45 patients ^c^ Significant difference between KIDSCREEN-52 filled in at start treatment and the group that filled in the KIDSCREEN-52 at start and end treatment (X2 (1) = 4.97; *p* = 0.026, V = 0.188)

### HRQoL at the start of treatment

Table 2 shows mean t-scores scored by children and adolescents on the KIDSCREEN-52 at the start of treatment, stratified for the DSM-5 criteria. Children with the diagnosis AN, BN and OSFED all had a lower HRQoL (≤ 0.5 SD of mean score) than the reference population for the dimensions Physical Well-being, Psychological Well-being, Moods and Emotions, Self-Perception, Autonomy, Financial Resources, Peers and Social Support, School Environment and Bullying. There were no statistically significant differences in t-scores between AN, BN and OSFED. This was confirmed with a Turkey’s post hoc test. Compared with the reference population the HRQoL in patients with ARFID was only lower for the dimension Physical Well-Being. For the dimensions Physical Well-being, Psychological Well-being, Moods and Emotions, Self-Perception, Autonomy, Parent Relations and Home Life and School Environment the t-scores of children with ARFID were significantly higher than those of the children who met criteria of all other eating disorders. Social Support and Peers was significantly higher in patients with ARFID compared to AN, but not with BN and OSFED. Univariate regression analysis in the group diagnosed with AN showed a significant association between a higher t-score on the domain Physical Well-being and higher BMI, BMI SD. Other variables were not associated with a higher or lower t-score.

**Table 2:** Mean t-score and Standard Deviation (SD) of KIDSCREEN-52 at Start of Treatment, Stratified into Different DSM-Criteria

	AN (*n* = 68)		BN (*n* = 13)		ARFID (*n* = 28)		OSFED (*n* = 29)	
Domain	Mean	(SD)	Mean	(SD)	Mean	(SD)	Mean	(SD)
Physical Well-being	**36.7**	(10.4)	**37.1**	(10.4)	**47.3**	(8.8)	**38.5**	(9.0)
Psychological Well-being	**36.8**	(9.2)	**33.5**	(9.1)	52.0	(9.1)	**37.0**	(9.6)
Moods and Emotions	**38.0**	(8.8)	**32.2**	(8.9)	54.5	(11.2)	**36.4**	(7.2)
Self-Perception	**37.6**	(7.7)	**31.4**	(7.9)	53.3	(9.9)	**35.0**	(9.2)
Autonomy	**44.9**	(8.8)	**43.2**	(8.7)	53.7	(9.2)	**45.1**	(5.5)
Parent Relations and Home Life	**46.9**	(9.9)	**43.5**	(9.8)	56.4	(8.8)	**44.0**	(9.8)
Financial Resources	55.5	(9.5)	54.1	(9.8)	51.4	(13.0)	56.0	(9.1)
Social Support and Peers	**41.8**	(10.9)	**43.8**	(10.6)	51.1	(11.5)	**44.0**	(7.6)
School Environment	**46.2**	(9.5)	**46.2**	(10.0)	57.0	(12.1)	**45.0**	(8.7)
Social Acceptance (Bullying)	48.0	(11.7)	46.4	(11.7)	47.7	(12.8)	48.0	(10.1)

Note.**Bold**: t-score lower than 0,5 SD compared with the mean t-score of the specific referential population; *table A7B-82* [25]

### HRQoL change between start and end of treatment

In Table 3 mean t-scores of the KIDSCREEN-52 at the start of treatment are compared with t-scores at the end evaluation. HRQoL showed a significant improvement in mean t-scores before and after treatment for Physical Well-being (t (46) = -4.4, *p* < 0.001), Psychological Well-being (t (45) = − 3.0, *P* = 0.004), Moods and emotions (t (45) = -3.3, *p* = 0.002) Self Perception (t (45) = -3.7, *p* = 0.001) and School environment (t (44) = -2.8, *p* = 0.008). However, after treatment the HRQoL for these dimensions did not normalize compared to normative reference values of Dutch children. The subgroup sample sizes were too small for findings relating to change in QoL before and after treatment to be stratified by diagnosis.

**Table 3:** Mean KIDSCREEN-52 t-score and Standard Deviation (SD) at Start Treatment and End-Evaluation

	KIDSCREEN-52 start treatment (*n* = 140)			KIDSCREEN-52 at start-end treatment (*n* = 47)					
Domain	Start treatment Mean (SD)			Start treatment Mean (SD)		End evaluation Mean (SD)		95% CI	*p*
Physical Well-being	**39.5**	(10.2)		**39.2**	(9.1)	**47.2**	(9.7)	-11.7– -4.3	0.000
Psychological Well-being	**40.2**	(10.9)		**40.4**	(10.3)	**45.6**	(10.9)	-8.6– -1.7	0.004
Moods and Emotions	**40.7**	(11.4)		**41.7**	(10.4)	**46.8**	(10.6)	-7.9– -1.9	0.002
Self-Perception	**39.7**	(11.2)		**40.1**	(9.6)	**46.0**	(10.6)	-8.6– -2.6	0.001
Autonomy	**46.9**	(8.9)		**46.6**	(7.7)	**48.1**	(7.9)	-3.6–1.2	0.330
Parent Relations and Home Life	**47.8**	(10.3)		48.7	(9.9)	50.6	(10.8)	-4.7–1.0	0.198
Financial Resources	54.0	(10.4)		55.4	(8.4)	55.7	(7.8)	-2.9 − 2.3	0.821
Social Support and Peers	**44.4**	(10.7)		**44.4**	(7.6)	**46.3**	(11.1)	-5.4–1.6	0.293
School Environment	**47.9**	(10.7)		48.7	(7.5)	53.4	(10.8)	-7.7– -1.2	0.008
Social Acceptance (Bullying)	48.3	(11.7)		49.6	(10.6)	51.3	(11.1)	-5.2–1.9	0.351

Note. **Bold**: t-score lower than 0.5 SD compared with the mean t-score of the specific referential population; *table A7B-82* [25]

## Discussion

This study shows that the self-reported HRQoL in children and adolescents receiving outpatient treatment in the Netherlands for an eating disorder is significantly lower on multiple dimensions at the beginning and end of treatment compared with the reference population. Most studies that have been conducted in children and adolescents are population-based studies that focus on disordered eating behaviours, yet they also show a significantly decreased mental HRQoL [9, 13, 29–34]. The study of Jenskins, showed similar results in a group of sixty-seven adolescents seeking treatment for an eating disorder [6].

The domain physical well-being is significantly lower for all types of eating disorders. This finding replicates that of Winkler et al. in which compared to the controls, adult women with AN had significantly impaired HRQoL as measured by the Eating Disorders Quality of Life (EDQOL) scale including lower physical functioning [35]. Yet several other studies showed only a significantly lower mental component summary and normal levels in the psychical component summary scored with Short Form-36 Health Survey (SF-36) [4, 6, 14, 36]. This difference could partially be explained by the use of different questionnaires, where some questionnaires could reflect the physical pathology of eating disorders rather than real physical health. The KIDSCREEN-52 for example specifically asked for fatigue, where other questionnaires ask for the ability to walk the stairs. When diagnosed with AN extensive exercise might be associated with the disease itself. Disease severity and duration of the eating disorder might also influence results. Children and adolescents in our study received one or more previous treatments in 55% of the patients and in 34% had a disease duration of more than one year. To gain more insight a univariate regression analysis was done in the group diagnosed with AN, which showed a significant association between a higher BMI and higher t-score on the domain Physical Well-being, suggesting that the results as shown within this study might be a reflection of real physical health rather than psychopathology.

When comparing AN, BN and OSFED this study does not find statistically significant differences similar as the meta-analysis on quality of life by Winkler et al. suggesting a similarity between these eating disorders with regard to HRQoL [35]. Notable exception to this are the children and adolescents with ARFID, who only score lower on the item Physical Well-being, unlike the children and adolescents classified with all other eating disorders who have lower scores on almost all HRQoL dimensions. This suggests that HRQoL affects children with ARFID differently. Hay et al. compared adults and adolescents from the age of 15 years with ARFID in the Australian population to other eating disorders and found, unlike the current study, a normal physical HRQoL and a significantly lower mental HRQoL [37]. A Dutch study by Krom et al., in which children were treated for ARFID in a Diagnostic Centre for Feeding Problems showed that the HRQoL, reported by their parents using TNO-AZL Preschool Children Quality of Life (TAPQOL) was significantly lower on the subscales appetite, lungs, stomach, motor functioning, and positive mood and liveliness, suggesting that both physical and mental HRQoL was affected [38]. The difference in mental HRQoL between the current study and the study by Krom et al. might be explained by an overestimation by parents of the child’s psychosocial functioning due to parent’s own concerns, and besides that it might be caused by age differences. Another explanation could be that ARFID differs from longer recognised disorders such as anorexia nervosa and bulimia nervosa in that they do not have a core psychopathology of body image disturbance or weight/shape overvaluation. Given that in adolescent and young adult women at least, it is clear that overvaluation of weight/shape is very strongly associated with impairment in quality of life including but not limited to the mental health domain, it is not too surprising that children and adolescents with a diagnosis of ARFID report relatively little impairment in mental HRQoL [39, 40]. The lower physical HRQoL that is seen in the current study might be explained by nutritional deficits often seen in children with ARFID [41].

The HRQoL shows significant improvement after treatment in all dimensions except for Autonomy and Social Support and Peers. However, HRQoL does not normalize compared to the reference population, and stays significantly impaired. This finding is consistent with considerations of other studies, namely that symptom remission alone is not sufficient for improvement in quality of life [42]. Studies looking at the long-term effects of eating disorders show that the long-term HRQoL after treatment continues to improve but is still not normalized after 8- or 30-years [14, 42–44]. Thus follow-up, with paying attention to HRQoL, should continue longer than the initial treatment. Similar to our results, greatest improvement in HRQoL was noted in the physical functioning domain [43, 44]. With childhood and adolescence being a critical period of development, the current study underlines the importance of treatment in which the success of the treatment is not based on BMI or amount of food intake alone, but focuses on other quality of life factors, such as psychological well-being, autonomy and social support.

There are limitations to this study. Due to the small subgroup sample size findings in the change in HRQoL before and after treatment could not be stratified by diagnosis. This study enrolled participants during a 14-year period, this longer period could have confounded the results due to changes in the care and treatments. Also, the retrospective nature of this study and the use of a generic HRQoL scale needs to be taken into consideration. Using generic HRQoL scales could give an over or underestimation of the HRQoL, since it does not focus specific on eating disorders, and questions for example about physical wellbeing could be an expression of the eating disorder rather than healthy behaviour. Our patients received both in- and outpatient treatment, which implies a certain disease severity and might not be generalizable to patients in other settings. HRQoL at the start of treatment could be lower or higher depending on the setting. Even though the children who completed the KIDSCREEN-52 only at the beginning of treatment and those who completed the questionnaire both at the start and end-evaluation are comparable, a large number of patients did not fill in the KIDSCREEN-52 at end-evaluation which might influence the outcome of quality of life after treatment, especially if the patients that did recover are the ones that did not fill in the questionnaire.

However, despite the limitations this descriptive study gives insight in the self-reported HRQoL of children and adolescents in the Netherlands treated for an eating disorder. It shows a significant reduction in both mental and physical HRQoL compared to the reference population with the exception of ARFID in which only physical HRQoL is impaired. This study also shows that even after treatment, children do not achieve normal HRQoL, which poses a potential risk to their development. Long-term follow-up of these children seems important, and more research is needed focusing on the effect of using quality of life parameters as most important measurements for recovery.

## Data Availability

No datasets were generated or analysed during the current study.

## Abbreviations

AN: Anorexia Nervosa
ARFID: Avoidant Restrictive Food Intake Disorder
BED: Binge Eating Disorder
BMI: Body Mass Index
BN: Bulimia Nervosa
DSM: Diagnostic and Statistical Manual of Mental Disorders
EDQOL: Eating Disorders Quality of Life
EDNOS: Eating disorder not otherwise specified
HRQoL: Health-related quality of life
SD: Standard deviation
SF: 36-Short Form-36 Health Survey
TAPQOL: TNO-AZL Preschool Children Quality of Life
